# Association between having a meal together with family and smoking: a cross-sectional nationwide survey

**DOI:** 10.1186/s12889-023-17155-9

**Published:** 2023-11-16

**Authors:** Gun Hee Cho, Yun Seo Jang, Jaeyong Shin, Chung-Mo Nam, Eun-Cheol Park

**Affiliations:** 1 Department of Social Policy Desk, Dong-A Ilbo, Seoul, Republic of Korea; 2 Department of Public Health, Graduate School, Yonsei University, Seoul, Republic of Korea; 3 Institute of Health Services Research, Yonsei University, Seoul, Republic of Korea; 4 Department of Preventive Medicine, Yonsei University College of Medicine, 50 Yonsei-Ro, Seodaemun-Gu, Seoul, 03722 Republic of Korea

**Keywords:** Smoking, Tobacco, Smoking cessation, Family meal, Family support, Cohesion

## Abstract

**Background:**

Smoking is a major risk factor that significantly affects public health. Although the South Korean government spends significant money on smoking cessation services, the smoking rate remains stagnant. Families influence health-conscious decisions, and family meals can positively affect smoking suppression and health behaviors. Therefore, this study investigated whether family meals are correlated with adults’ smoking behaviors.

**Methods:**

This study used data from the 2019–2021 Korean National Health and Nutrition Examination Survey. Having a meal together with family was defined as “yes” for those who have at least one meal with their family each day and “no” for those who do not. Current smoking status was classified as having smoked at least 5 packs of cigarettes (100 cigarettes) in one’s lifetime and having used either conventional cigarettes or e-cigarettes in the last 30 days. Multiple logistic regression analyses were used to examine the association between eating together, smoking, and weight application.

**Results:**

When comparing the group that ate with their family compared to the group that did not, the odds ratio for current smoking status was 1.27 (95% confidence interval [CI]: 1.05–1.54) for male participants and 1.90 (95% CI: 1.33–2.71) for female participants. This showed a dose-dependent effect according to the frequency of family meals. Those who smoked conventional cigarettes had a strong association (men: OR 1.28, 95% CI 1.00–1.67; women: OR 2.22, 95% CI 1.42–3.46). However, those who only vaped e-cigarettes or used both conventional cigarettes and e-cigarettes had no statistically significant correlations.

**Conclusion:**

This study provides evidence suggesting that eating meals as a family is related to smoking behavior and can positively affect smoking cessation intentions in adults. Consequently, a smoking cessation program can be developed that uses social support, such as encouraging family meals.

**Supplementary Information:**

The online version contains supplementary material available at 10.1186/s12889-023-17155-9.

## Background

Smoking is a global public health issue and the most significant risk factor affecting health. The World Health Organization (WHO) estimates that the annual death toll from smoking is 8 million, and has rated tobacco as “one of the biggest public health threats the world has ever faced” [1]. Smoking is recognized as a risk factor for all types of cancer [2]; cardiovascular diseases such as ischemic heart disease [3, 4], arrhythmia [5, 6], and stroke [3, 7]; respiratory diseases including chronic obstructive pulmonary disease [8]; and mental disorders such as depression [9] and schizophrenia [9]. In Korea, the conventional cigarette smoking rate has decreased among adults. In 2021, the smoking rate of conventional cigarettes among adults was 19.3%, a decrease of 3% from 22.3% in 2017. However, the magnitude of this decline varies significantly by sex and has not yet reached the government’s target [10]. According to the government’s 5th National Health Plan (Health Plan 2021 ~ 2030), the smoking rate for adult men and women will reach 25.0% and 4.0% by 2030, compared to 36.7% for men and 7.5% for women in 2018. Additionally, there has been an increase in the use of e-cigarettes instead of conventional cigarettes [11]. The WHO Health Organization emphasizes that the health benefits of smoking cessation are as evident as the harmful effects of smoking [1]. Previous studies have shown that the risk of ischemic heart disease [12], acute myocardial infarction [13], and lung cancer [12] mortality are reduced in people with a long smoking cessation period. Therefore, smoking cessation is considered a worldwide major public health priority and efforts are made annually to set targets to reduce smoking rates.

Family is the most important and central experience in social relationships [14]. According to previous studies, individuals who live with their families and have a strong sense of kinship often make health-promoting decisions [15]. In particular, the odds ratio (OR) for attempting to quit smoking was higher among those who were married and cohabiting with their spouse [16]. This can be attributed to interactions, communication, and conversations, such as family members expressing dissatisfaction with second-hand smoke or concern for the family’s health, which positively influences smokers’ intentions to quit [16]. In other words, family members’ interactions and influence on health management can positively affect smokers. Furthermore, several studies have shown that dining with someone has a positive impact on mental health [17, 18]. Having meals with family members has a positive effect on family cohesion, facilitating easier control over health behaviors within the household and yielding beneficial outcomes [19, 20].

Previous studies on the association between family meals and smoking have predominantly focused on adolescents, both domestically and internationally. Cohort studies conducted by the National Heart, Lung, and Blood Institute in the United States and studies on Minnesota adolescents indicated that dining with family members increases the sense of kinship within the family and ultimately inhibits smoking behavior through the structure and flow of family dynamics [21–23]. Moreover, based on data like the National Longitudinal Survey of Youth in the U.S., studies have analyzed the correlation between the frequency of family meals among adolescents and smoking [24]. Similar findings have been observed in Israel and Scotland [25, 26]. Studies conducted in Korea have also shown that the OR of smoking experience among groups of adolescents who do not have meals with their families is higher than those who have meals with their families, and research results indicated that a positive atmosphere during family meals reduces problematic behaviors in adolescents [27]. A previous study targeting middle-aged men revealed that higher satisfaction with family relationships was associated with lower odds of smoking [28]. Obtaining advice and support within the family enhances smoking cessation behavior, while tension within the family increases the motivation for smoking as a way to relieve stress [29].

Therefore, this study aimed to investigate the association between having meals with family members and smoking behavior in adults, and identify practical information that can be used to suggest policies and establish institutional support to enhance smoking cessation success rates.

## Methods

### Research design

This study used cross-sectional data from the 2019–2021 National Health and Nutrition Examination Survey (KNHANES), which was conducted by the Korea Centers for Disease Control and Prevention Agency (KDCA). The KNHANES is an annual self-reported survey designed to assess the health and nutritional status of South Koreans of all ages, utilizing a stratified, multi-stage, cluster sampling methodology. Therefore, the survey was conducted using random cluster sampling, allowing for statistical generalization of the research findings to the general population. According to the National Health Promotion Act 16, the KNHANES is a nationwide survey that calculates national statistics through the health level, health-related consciousness and behavior, and food and nutrition intake of about 10,000 people aged 1 or older. The survey was introduced in 1998 and conducted every three years until 2005 and has been conducted annually since 2007. Anyone can access it, and we conducted an analysis using secondary data. It provides information on the development and assessment of health policies and programs. Additionally, the results of the KNHANES are used to compare health status between countries, as required by organizations such as the World Health Organization (WHO) and the Organisation for Economic Co-operation and Development (OECD). This study was exempt from the ethics review board because the KNHANES adheres to the Declaration of Helsinki.

### Study population

Of the 22,559 participants in the survey, those aged less than 19 and those who did not participate in the KNHANES smoking questionnaire were excluded (*n* = 3,868). Additionally, participants who lived alone (i.e., single-person households; *n* = 2,628), and those with missing data (*n* = 4,984) were excluded. Consequently, a final sample of 11,079 participants was included in this study, as presented in Supplementary Table 1.

### Variables

The main dependent variable was current smoking status. In this study, current smoking status was categorized as “currently smoking” if the participant reported using either conventional cigarettes or electronic cigarettes (e-cigarettes). Specifically, individuals who had smoked more than five packs (100 cigarettes) in their lifetime and had been smoking conventional cigarettes or using e-cigarettes in the last 30 days were classified as “currently smoking,” while those who did not meet these criteria were classified as “currently non-smoking.” The current use of e-cigarettes has been defined as a question of whether cartridge-type or liquid-type e-cigarettes are currently used. People who formerly smoked were classified as those who smoked more than 5 packs (100 cigarettes) in their lifetime but did not smoke at present. This categorization is consistent with previous studies that investigated smoking behavior using the same research tool [30–33].

The main independent variable was whether the participant had a meal with family members and was defined using two survey questions. The first question was “In the last year, have you eaten with others when having a meal?” and only those who answered “yes” to the first question were asked a second question. The second question was, “Who was the person you had a meal with?”. This question could be answered as “family” or “non-family.” Accordingly, we classified those who answered “family” as the group who had a meal with family and those who answered “non-family” as the group who did not have a meal with family. The questions were asked separately for breakfast, lunch, and dinner, and those who had at least one family meal daily were categorized as “yes,” while those who did not were categorized as “no.” In addition, we measured the frequency of family meals per day by combining questions based on whether family meals (each breakfast, lunch, and dinner) occurred or not.

The covariates included demographic (gender, age, and region), socioeconomic (marital status, number of family members, household income, educational level, household generation composition, and occupational categories), health-related (body mass index [BMI], drinking status, physical activity, and number of chronic diseases), and other factors (frequency of eating out and survey year). Specifically, occupational categories refer to office workers as “white collar,” production workers as “blue collar,” and service workers who provide or sell services as “pink collar.”

### Statistical analysis

Weighted estimates were used in all analyses to improve the representativeness and generalizability of the data, and clusters and strata were assigned to the study population. Briefly, we used variables of stratified sampling (kstrata) and clustering (primary sampling units) provided by KNHANES to explain the limited proportion of the final population. Descriptive analysis was used to determine the general characteristics of the study group, including frequencies (*N*) and percentages (*%)*, and the results were assessed and compared using chi-squared tests. Following this, multiple logistic regression analysis was performed, controlling for covariates, to examine the association between current smoking status and having meals with family. Subgroup analyses, stratified by independent variables, were performed according to marital status, educational level, region, occupational category, household generation composition, and number of household members. Furthermore, a subgroup analysis was performed for a more complete analysis, stratified by dependent variables (i.e., smoking behavior, cigarette type, and attempt or plan for smoking cessation) and confounding variables (i.e., type and frequency of having family meals). Specifically, attempt or plan for smoking cessation was measured by the questions, “Have you ever quit smoking for more than a day (24 h) in the past year?” and “Do you have any plans to quit smoking in the next month?” All analyses were stratified by gender to account for gender differences in conventional or e-cigarette use, which was more prevalent in male participants. Statistical analyses were performed using SAS version 9.4 (SAS Institute Inc., Cary, NC, USA).

## Results

Characteristics of the study population are presented in Table 1 and descriptive statistics for each smoking behavior of participants are shown in Supplementary Table 2. Among male participants, 3,855 (80.3%) reported having meals with their families, whereas 5,211 (83.0%) female participants reported having meals with their families. Among male participants who had meals with their families, those who currently smoke accounted for 29.6%. Among those who did not have meals with their families, the current smoking rate was 38.0%. Similarly, among female participants, the current smoking rate was 4.0% among those who had meals with their families, while the current smoking rate was 9.2% among those who did not have meals with their families. The chi-squared test revealed a statistically significant association between having a meal with family and current smoking status for both male and female participants (*p* < 0.0001).

**Table 1:** General characteristics of the study population

Variables	Current smoking status													
	**Male**						***P-value***	**Female**						***P-value***
	**Total**		**Yes**		**No**			**Total**		**Yes**		**No**		
	**N**	**%**	**N**	**%**	**N**	**%**		**N**	**%**	**N**	**%**	**N**	**%**	
**Total (*N* = 11,079)**	4,800	100.0	1,501	31.3	3,299	68.7		6,279	100.0	307	4.9	5,972	95.1	
**Having a meal together with family**							< .0001							< .0001
Yes	3,855	80.3	1,142	29.6	2,713	70.4		5,211	83.0	209	4.0	5,002	96.0	
No	945	19.7	359	38.0	586	62.0		1,068	17.0	98	9.2	970	90.8	
**Age**							< .0001							< .0001
19–29	627	13.1	208	33.2	419	66.8		733	11.7	76	10.4	657	89.6	
30–39	627	13.1	254	40.5	373	59.5		904	14.4	76	8.4	828	91.6	
40–49	882	18.4	360	40.8	522	59.2		1,287	20.5	70	5.4	1,217	94.6	
50–59	853	17.8	310	36.3	543	63.7		1,349	21.5	36	2.7	1,313	97.3	
60-	1,811	37.7	369	20.4	1,442	79.6		2,006	31.9	49	2.4	1,957	97.6	
**Marital status**							< .0001							< .0001
Married	3,755	78.2	1,098	29.2	2,657	70.8		4,737	75.4	193	4.1	4,544	95.9	
Divorced, Separated	937	19.5	335	35.8	602	64.2		1,266	20.2	87	6.9	1,179	93.1	
Single, widow	108	2.3	68	63.0	40	37.0		276	4.4	27	9.8	249	90.2	
**Household income**							0.0001							0.0026
Low	641	13.4	156	24.3	485	75.7		809	12.9	31	3.8	778	96.2	
Mid-low	1,167	24.3	371	31.8	796	68.2		1,552	24.7	93	6.0	1,459	94.0	
Mid-high	1,380	28.8	475	34.4	905	65.6		1,865	29.7	106	5.7	1,759	94.3	
High	1,612	33.6	499	31.0	1,113	69.0		2,053	32.7	77	3.8	1,976	96.2	
**Educational level**							< .0001							< .0001
Middle school or below	1,035	21.6	267	25.8	768	74.2		1,687	26.9	69	4.1	1,618	95.9	
High school	1,714	35.7	615	35.9	1,099	64.1		2,130	33.9	157	7.4	1,973	92.6	
University or beyond	2,051	42.7	619	30.2	1,432	69.8		2,462	39.2	81	3.3	2,381	96.7	
**Region**							0.2532							0.5669
Metropolitan	2,043	42.6	665	32.6	1,378	67.4		2,835	45.2	145	5.1	2,690	94.9	
Urban	1,737	36.2	529	30.5	1,208	69.5		2,285	36.4	112	4.9	2,173	95.1	
Rural	1,020	21.3	307	30.1	713	69.9		1,159	18.5	50	4.3	1,109	95.7	
**Occupational categories**							< .0001							0.1121
White	1,367	28.5	413	30.2	954	69.8		1,500	23.9	73	4.9	1,427	95.1	
Pink	489	10.2	197	40.3	292	59.7		954	15.2	61	6.4	893	93.6	
Blue	1,584	33.0	548	34.6	1,036	65.4		890	14.2	43	4.8	847	95.2	
Inoccupation	1,360	28.3	343	25.2	1,017	74.8		2,935	46.7	130	4.4	2,805	95.6	
**Physical activity**							0.0136							0.2553
Adequate	2,221	46.3	655	29.5	1,566	70.5		2,568	40.9	116	4.5	2,452	95.5	
Inadequate	2,579	53.7	846	32.8	1,733	67.2		3,711	59.1	191	5.1	3,520	94.9	
**Current drinking status**							< .0001							< .0001
Never or occasionally	958	20.0	171	17.8	787	82.2		2,231	35.5	55	2.5	2,176	97.5	
2 ~ 4 times / month	2,320	48.3	721	31.1	1,599	68.9		3,373	53.7	157	4.7	3,216	95.3	
2 ~ 4 times / week	1,522	31.7	609	40.0	913	60.0		675	10.8	95	14.1	580	85.9	
**BMI**							0.5744							0.1402
Normal and underweight	1,463	30.5	469	32.1	994	67.9		3,134	49.9	149	4.8	2,985	95.2	
Overweight	1,265	26.4	382	30.2	883	69.8		1,282	20.4	53	4.1	1,229	95.9	
Obese	2,072	43.2	650	31.4	1,422	68.6		1,863	29.7	105	5.6	1,758	94.4	
**Number of chronic diseases**							0.0006							< .0001
0	2,372	49.4	793	33.4	1,579	66.6		3,512	55.9	210	6.0	3,302	94.0	
1	1,454	30.3	448	30.8	1,006	69.2		1,598	25.4	58	3.6	1,540	96.4	
≥ 2	974	20.3	260	26.7	714	73.3		1,169	18.6	39	3.3	1,130	96.7	
**Household generation composition**							< .0001							0.1859
1st generation	1,662	34.6	403	24.2	1,259	75.8		1,789	28.5	85	4.8	1,704	95.2	
2nd generation	2,876	59.9	1,004	34.9	1,872	65.1		4,024	64.1	191	4.7	3,833	95.3	
3rd generation or more	262	5.5	94	35.9	168	64.1		466	7.4	31	6.7	435	93.3	
**Number of household members**							< .0001							0.0432
2	1,920	40.0	518	27.0	1,402	73.0		2,325	37.0	120	5.2	2,205	94.8	
3	1,354	28.2	449	33.2	905	66.8		1,815	28.9	97	5.3	1,718	94.7	
4	1,147	23.9	396	34.5	751	65.5		1,562	24.9	56	3.6	1,506	96.4	
≥ 5	379	7.9	138	36.4	241	63.6		577	9.2	34	5.9	543	94.1	
**Frequency of eating out**							< .0001							0.0004
Everyday	1,367	28.5	496	36.3	871	63.7		861	13.7	55	6.4	806	93.6	
1 times more / week	2,335	48.6	723	31.0	1,612	69.0		3,339	53.2	182	5.5	3,157	94.5	
1 times more / month	785	16.4	209	26.6	576	73.4		1,496	23.8	45	3.0	1,451	97.0	
Never or less than once a month	313	6.5	73	23.3	240	76.7		583	9.3	25	4.3	558	95.7	
**Year**							0.1814							0.9163
2019	1,840	38.3	599	32.6	1,241	67.4		2,406	38.3	115	4.8	2,291	95.2	
2020	1,482	30.9	465	31.4	1,017	68.6		1,940	30.9	98	5.1	1,842	94.9	
2021	1,478	30.8	437	29.6	1,041	70.4		1,933	30.8	94	4.9	1,839	95.1	

Table 2 presents the results of the multiple logistic regression analysis, adjusting for all covariates and stratified by gender, to examine the association between having a meal with family and current smoking status. The odds of currently smoking among male participants were 1.27 (95% confidence interval [CI]: 1.05–1.54) if they did not have a meal with family compared to those who did. Among female participants, the odds of currently smoking were 1.90 (95% CI: 1.33–2.71) if they did not have a meal with family compared to those who did.

**Table 2:** Results of factors associated between having a meal together with family and smoking

Variables	Current smoking status							
	**Male**				**Female**			
	**OR**	**95% CI**			**OR**	**95% CI**		
**Having a meal together with family**								
Yes	1.00				1.00			
No	1.27	(1.05	-	1.54)	1.90	(1.33	-	2.71)
**Age**								
19–29	2.04	(1.37	-	3.02)	25.02	(11.62	-	53.88)
30–39	3.26	(2.33	-	4.56)	17.86	(8.74	-	36.49)
40–49	2.88	(2.14	-	3.88)	8.33	(4.16	-	16.67)
50–59	2.07	(1.56	-	2.73)	1.77	(0.93	-	3.39)
60-	1.00				1.00			
**Marital status**								
Married	1.00				1.00			
Divorced, Separated	1.08	(0.80	-	1.47)	0.63	(0.37	-	1.06)
Single, widow	2.72	(1.65	-	4.50)	1.64	(0.87	-	3.12)
**Household income**								
Low	1.11	(0.79	-	1.54)	1.18	(0.64	-	2.17)
Mid-low	1.18	(0.95	-	1.48)	1.42	(0.97	-	2.07)
Mid-high	1.22	(1.01	-	1.48)	1.46	(1.01	-	2.11)
High	1.00				1.00			
**Educational level**								
Middle school or below	1.28	(0.95	-	1.70)	6.70	(3.68	-	12.22)
High school	1.34	(1.10	-	1.64)	3.44	(2.34	-	5.06)
University or beyond	1.00				1.00			
**Region**								
Metropolitan	1.20	(0.95	-	1.51)	1.51	(0.98	-	2.31)
Urban	1.01	(0.80	-	1.27)	1.44	(0.94	-	2.22)
Rural	1.00				1.00			
**Occupational categories**								
White	1.00				1.00			
Pink	1.38	(1.05	-	1.82)	0.93	(0.58	-	1.48)
Blue	1.37	(1.09	-	1.72)	1.01	(0.58	-	1.77)
Inoccupation	1.17	(0.91	-	1.50)	0.93	(0.62	-	1.38)
**Physical activity**								
Adequate	1.00				1.00			
Inadequate	1.25	(1.07	-	1.45)	1.28	(0.94	-	1.73)
**Current drinking status**								
Never or occasionally	1.00				1.00			
2 ~ 4 times / month	1.91	(1.51	-	2.42)	1.70	(1.14	-	2.53)
2 ~ 4 times / week	3.13	(2.43	-	4.03)	4.67	(3.06	-	7.13)
**BMI**								
Normal and underweight	1.00				1.00			
Overweight	0.90	(0.73	-	1.12)	0.92	(0.58	-	1.44)
Obese	0.88	(0.74	-	1.04)	1.34	(0.93	-	1.94)
**Number of chronic diseases**								
0	1.00				1.00			
1	1.16	(0.97	-	1.38)	0.93	(0.63	-	1.39)
≥ 2	1.10	(0.86	-	1.39)	0.99	(0.57	-	1.74)
**Household generation composition**								
1st generation	1.00				1.00			
2nd generation	1.02	(0.71	-	1.47)	0.75	(0.44	-	1.30)
3rd generation or more	1.06	(0.62	-	1.81)	1.55	(0.71	-	3.35)
**Number of household members**								
2	1.00				1.00			
3	0.88	(0.63	-	1.22)	0.89	(0.53	-	1.48)
4	0.89	(0.62	-	1.27)	0.44	(0.24	-	0.79)
≥ 5	0.98	(0.63	-	1.51)	0.43	(0.23	-	0.83)
**Frequency of eating out**								
Everyday	1.00				1.00			
1 times more / week	0.94	(0.80	-	1.12)	0.94	(0.62	-	1.41)
1 times more / month	0.99	(0.76	-	1.28)	0.97	(0.55	-	1.70)
Never or less than once a month	1.03	(0.70	-	1.51)	1.57	(0.77	-	3.20)
**Year**								
2019	1.00				1.00			
2020	0.95	(0.79	-	1.13)	0.97	(0.67	-	1.40)
2021	0.97	(0.82	-	1.16)	1.08	(0.74	-	1.57)

Table 3 presents the subgroup analysis performed to evaluate the combined effect of having a meal together with family, age, marital status, educational level, region, occupational categories, household generation composition, and number of household members on current smoking status. Regarding male participants who did not have meals with their family, the strongest association with current smoking status was observed among the older adult population (60 + years of age: OR 1.79, 95% CI 1.22–2.62), married group (OR 1.25, 95% CI 1.00–1.57), and inoccupation group (OR 1.87, 95% CI 1.19–2.93). Regarding female participants who did not have meals with their family, an association with current smoking status was observed among middle-aged adults (30–39 years of age: OR 2.10, 95% CI 1.02–4.31; 40–49 years of age: OR 2.07, 95% CI 1.02–4.18), unmarried status (divorced or separated: OR 2.12, 95% CI 1.20–3.73; single or widowed: OR 8.93, 95% CI 1.71–46.62), and individuals in pink or blue-collar occupations (pink-collar: OR 2.42, 95% CI 1.20–4.88; blue-collar: OR 3.55, 95% CI 1.56–8.06). Additionally, for male participants, an association was observed with lower education levels (OR 1.84, 95% CI 1.14–2.95), rural region (OR 2.00, 95% CI 1.38–2.92), 1st household generation composition (OR 1.59, 95% CI 1.07–2.36), and two household members (OR 1.59, 95% CI 1.13–2.23). For female participants, the same association was shown with low education levels (OR 2.12, 95% CI 1.08–4.17]; rural region (OR 4.37, 95% CI 1.70–11.25); 1st household generation composition (OR 1.88, 95% CI 0.93–3.81); and two household members (OR 2.11, 95% CI 1.22–3.66). A linear trend was evident in ORs in accordance with these factors.

**Table 3:** Results of subgroup analysis stratified by independent variables

**Variables**^a^	**Current smoking status**									
	**Male**					**Female**				
	**Yes**	**No**				**Yes**	**No**			
	**OR**	**OR**	**95% CI**			**OR**	**OR**	**95% CI**		
**Age**										
19–29	1.00	1.19	(0.72	-	1.96)	1.00	1.80	(0.93	-	3.52)
30–39	1.00	0.81	(0.50	-	1.32)	1.00	2.10	(1.02	-	4.31)
40–49	1.00	1.15	(0.78	-	1.70)	1.00	2.07	(1.02	-	4.18)
50–59	1.00	1.38	(0.91	-	2.09)	1.00	0.73	(0.25	-	2.14)
60-	1.00	1.79	(1.22	-	2.62)	1.00	1.53	(0.66	-	3.55)
**Marital status**										
Married	1.00	1.25	(1.00	-	1.57)	1.00	1.48	(0.88	-	2.51)
Divorced, Separated	1.00	1.17	(0.78	-	1.74)	1.00	2.12	(1.20	-	3.73)
Single, Widow	1.00	3.44	(0.50	-	23.77)	1.00	8.93	(1.71	-	46.62)
**Educational level**										
Middle school or below	1.00	1.84	(1.14	-	2.95)	1.00	2.12	(1.08	-	4.17)
High school	1.00	1.22	(0.89	-	1.67)	1.00	1.83	(1.12	-	2.98)
University or beyond	1.00	1.19	(0.91	-	1.56)	1.00	1.72	(0.94	-	3.14)
**Region**										
Metropolitan	1.00	1.19	(0.89	-	1.57)	1.00	1.28	(0.83	-	1.97)
Urban	1.00	1.25	(0.92	-	1.71)	1.00	2.33	(1.26	-	4.32)
Rural	1.00	2.00	(1.38	-	2.92)	1.00	4.37	(1.70	-	11.25)
**Occupational categories**										
White	1.00	1.12	(0.80	-	1.56)	1.00	1.87	(0.92	-	3.81)
Pink	1.00	1.26	(0.71	-	2.24)	1.00	2.42	(1.20	-	4.88)
Blue	1.00	1.13	(0.80	-	1.58)	1.00	3.55	(1.56	-	8.06)
Inoccupation	1.00	1.87	(1.19	-	2.93)	1.00	1.68	(0.95	-	2.98)
**Household generation composition**										
1st generation	1.00	1.59	(1.07	-	2.36)	1.00	1.88	(0.93	-	3.81)
2nd generation	1.00	1.25	(0.99	-	1.57)	1.00	1.81	(1.15	-	2.84)
3rd generation or more	1.00	0.71	(0.30	-	1.69)	1.00	1.67	(0.52	-	5.41)
**Number of household members**										
2	1.00	1.59	(1.13	-	2.23)	1.00	2.11	(1.22	-	3.66)
3	1.00	1.34	(0.97	-	1.86)	1.00	1.51	(0.89	-	2.56)
4	1.00	1.17	(0.83	-	1.66)	1.00	1.20	(0.94	-	2.14)
≥ 5	1.00	0.77	(0.35	-	1.69)	1.00	1.05	(0.32	-	3.45)

^a^Adjusted for all covariates (age, marital status, educational level, region, occupational categories, household generation composition, and number of household members)

**Fig. 1:**
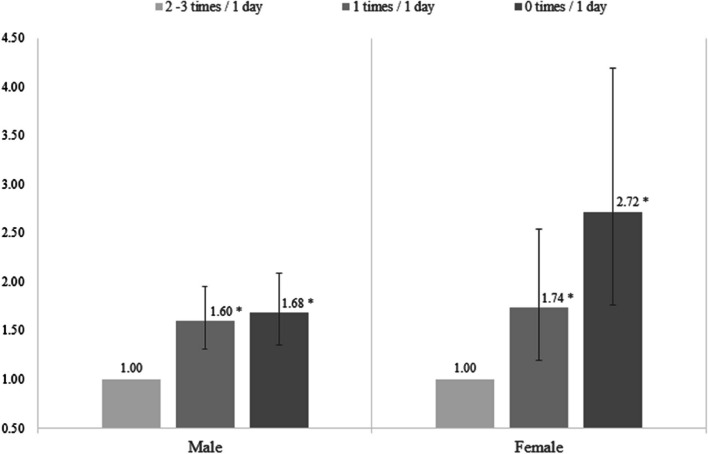
Results of subgroup analysis stratified by frequency of having a meal together with family (Error bars: 95% confidence interval. *p*-value < .05; All covariates are adjusted

Figure 1 presents the results of the subgroup analysis stratified by gender, indicating the association between the frequency of having a meal with family and smoking status. When considering individuals who had meals with their families as the reference category, both genders showed a linear increase in the ORs for current smoking status as the frequency of shared meals decreased (1 time per day—male: OR 1.60, 95% CI 1.31–1.95; female: OR 1.74, 95% CI 1.19–2.54 / 0 times per day—male: OR 1.68, 95% CI 1.36–2.09; female: OR 2.72, 95% CI 1.77–4.19). Furthermore, when analyzing the relationship based on meal types (Table 4), the odds of current smoking were higher when male participants did not have breakfast or lunch together (not having breakfast together: OR 1.62, 95% CI 1.36–1.92; not having lunch together: OR 1.50, 95% CI 1.23–1.83), while for female participants, the odds were higher when they did not have breakfast or dinner together (not having breakfast together: OR 1.98, 95% CI 1.38–2.84; not having dinner together: OR 1.99, 95% CI 1.42–2.77).

**Table 4:** Results of subgroup analysis stratified by type of having a meal together with family

**Variables**^a^	**Current smoking status**							
	**Male**				**Female**			
	**OR**	**95% CI**			**OR**	**95% CI**		
**Type of having a meal**								
Having together	1.00				1.00			
Not having breakfast	1.62	(1.36	-	1.92)	1.98	(1.38	-	2.84)
Not having lunch	1.50	(1.23	-	1.83)	1.37	(0.90	-	2.07)
Not having dinner	1.17	(0.98	-	1.39)	1.99	(1.42	-	2.77)

^a^Adjusted for all covariates (age, marital status, educational level, region, occupational categories, household generation composition, and number of household members)

**Fig. 2:**
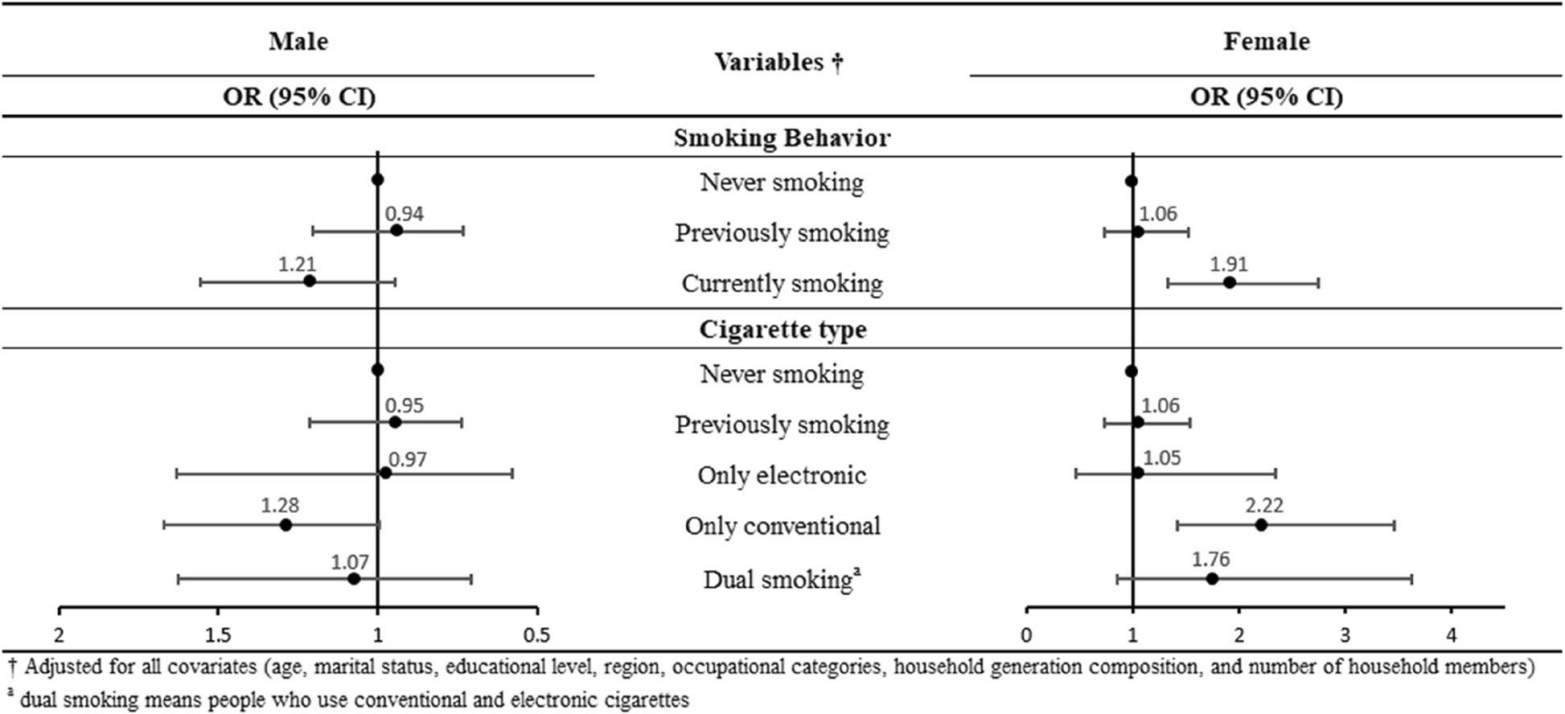
Results of subgroup analysis stratified by smoking behavior and cigarette type

Figure 2 presents the analysis of the association between having a meal with family and current smoking status as well as past smoking experience, types of tobacco products used, and smoking cessation attempts or plans among people who currently smoke. Generally, when not having a meal with family, people who currently smoke had higher ORs than people who previously smoked (male: OR 1.21, 95% CI 0.94–1.56; female: OR 1.91, 95% CI 1.33–2.74), and showed a strong statistical association with smoking only conventional cigarettes (male: OR 1.28, 95% CI 1.00–1.67; female: OR 2.22, 95% CI 1.42–3.46). In contract, dual smoking (male: OR 1.07, 95% CI 0.71–1.63; female: OR 1.76, 95% CI 0.86–3.62) and e-cigarette-only use (male: OR 0.97, 95% CI 0.58–1.63; female: OR 1.05, 95% CI 0.47–2.34) showed a relatively low association with family meals. Additionally, as shown in Table 5, groups that did not have meals with their families tended to have high ORs for currently having no history of smoking cessation attempts (male: OR 1.06, 95% CI 0.80–1.41) in the past year and not planning to quit smoking in the future (male: OR 1.38, 95% CI 1.02–1.86; female: OR 1.71, 95% CI 0.70–4.19).

**Table 5:** Subgroup analysis of smoking cessation attempt and plan among only people who currently smoke

Variables^a^	Having a meal together with family (Ref = 'Yes')							
	**Male**				**Female**			
	**OR**	**95% CI**			**OR**	**95% CI**		
**Smoking cessation attempt**								
Yes	1.00				1.00			
No	1.06	(0.80	-	1.41)	0.87	(0.39	-	1.91)
**Smoking cessation plan**								
Yes	1.00				1.00			
No	1.38	(1.02	-	1.86)	1.71	(0.70	-	4.19)

^a^Adjusted for all covariates (age, marital status, educational level, region, occupational categories, household generation composition, and number of household members)

## Discussion

The results showed that both genders had a lower risk of current smoking when eating with their families than when they did not. This is in line with previous studies showing that having a meal with families has a positive association with increasing health behaviors. This trend was more pronounced among female participants than male participants. According to previous studies, women who smoke are more emotionally affected than men who smoke when deciding to quit smoking, and the effect of nicotine therapy replacement is relatively low [19, 24, 27]. Considering this, it can be inferred that family meals plays a more important role in smoking cessation among female participants than male participants [34].

It is particularly noteworthy that the risk of smoking was lower in the group that often had meals with their families. Both men and women were less likely to smoke when they ate at least one meal a day with their families than the group who ate alone, and both men and women were less likely to smoke in the group that ate more than two meals a day with their families; both were statistically significant while controlling for other covariates or confounding variables such as age and socioeconomic status. This supports previous studies showing that the more frequent family meals are, the lower the likelihood of smoking among male and female adolescents [24]. Additionally, another study on middle-aged men found that high family relationship satisfaction lowered the risk of smoking and suggested that family advice may have strengthened smoking cessation behavior [29]. The present study’s findings support those of previous studies and show an association between family meals and smoking in adults, both men and women, and between adolescents and middle-aged men [24, 29]. Furthermore, even when the three meals (breakfast, lunch, and dinner) were analyzed separately, the ORs for current smoking status, regardless of the three meals with family, were lower. However, it is assumed that the reason lunch was not statistically significant is that the number of samples analyzed was insufficient due to participants often not spending time with their families in the age group with an active social life.

According to the results of the independent subgroup analysis, the age groups with a relatively higher association between having a meal together with family and smoking were male participants in their 60 s and older and female participants in their 30 s and 40 s. According to previous studies, it can be assumed that older adult men’s family ties play an important role in health care [35, 36]. Additionally, the fact that women in their 30 s and 40 s constitute an age group that focuses on pregnancy, childbirth, and childcare may have increased the relationship between family ties and smoking [37–40]. In addition, smoking cessation programs using family support could be more active in rural than metropolitan or urban areas, and the need to be actively implemented in two-person households, which are simple households, was emphasized.

Finally, the analysis conducted by dividing the relationship between having a meal with family and smoking by cigarette type revealed remarkable results. The group that smoked only conventional cigarettes was the most affected by family meals. People who used both conventional cigarettes and e-cigarettes were relatively less affected, and the group that used only e-cigarettes had the lowest association. Because e-cigarettes smell relatively less than conventional cigarettes, e-cigarette users are presumed to have fewer opportunities to receive health advice from families while eating meals together [41, 42]. Further, the subgroup analysis results of currently smoking people’s attempts or plans to quit smoking showed that women who had a meal with family are likely to have attempted to quit smoking within the one year. Moreover, they were more likely to plan to quit smoking the following month. This is in line with a previous study [34], and it is suggested that currently smoking women who have meals with their family have a relative intention to quit smoking due to emotional effects such as family pressure to induce smoking cessation.

This study has several limitations. First, this study was cross-sectional, which means that the temporal relationship is unclear, and reverse causality may be possible. Regarding family meals, it is unknown when the habit of eating with the family started and whether it preceded smoking initiation. Therefore, caution is required when interpreting these results and further prospective cohort studies are required to clarify these findings. Second, the KNHANES uses self-report surveys, which introduce potential limitations in the reliability and accuracy of health-related, socioeconomic, and smoking statuses. This can result in recall bias, particularly regarding the underestimation of smoking prevalence. Third, despite attempts to include as many independent variables related to family meals and smoking as possible, potentially uncontrolled confounding variables may still exist. Fourth, it is important to note that the presence or absence of family meals does not necessarily reflect the depth of family relationships or the frequency of face-to-face interactions. It is possible for families to have a close bond even without regularly eating meals together, or conversely, not to have a strong bond despite sharing meals together. Finally, this study did not assess the quality of family meals. According to a previous study, even when families eat together, there can be significant differences in the proportion of mealtime spent in conversation depending on family members’ participation [25]. In this study, only the presence, frequency, and types of family meals were analyzed, while, due to limitations in the KNHANES data, data on the number of family members participating in meals or the extent of conversation with families during meals were not collected.

Despite these limitations, this study has several strengths. First, this study utilized data from the KNHANES, a nationally representative survey that reflects the health behaviors and characteristics of South Koreans. Second, the inclusion of recent data from 2019 to 2021 is significant, as it encompasses not only current smoking status but also factors such as e-cigarette use, past smoking history, and smoking cessation attempts and plans. Third, although previous studies examining the association between family meals and smoking have often focused only on conventional cigarette smoking or adolescents, this study included all adults and e-cigarette smoking.

## Conclusion

This study found that having a meal with family members may have a positive effect on adult smoking control and smoking cessation intention. Considering tobacco addiction and the continued release of new e-cigarettes, the government needs to actively utilize social support for smoking cessation activities, such as encouraging having meals with family members. Additionally, educational programs that promote family dialogue and remind people who smoke of their bonds with their families are important. Thus, it is necessary to develop programs and actively promote smoking cessation clinics at public health centers and educate family members about the importance of family meals. By combining these efforts, the health of people who smoke can be improved and a healthy culture of smoking cessation can be created through family and social support.

### Supplementary Information

**Additional file 1: Supplementary 1. **Flowchart of the study participants displaying the inclusion and exclusion.

**Additional file 2: Supplementary 2.** General characteristics of the study population for each smoking behavior (cigarette type).

**Additional file 3: Supplementary 3. **Subgroup analysis of smoking cessation attempt and plan among all participants.

## Data Availability

The datasets generated and/or analyzed during the current study are available in the Korea National Health and Nutrition Examination Survey (KNHANES) 2020, https://knhanes.kdca.go.kr/knhanes/sub03/sub03_02_05.do

## Abbreviations

OR: Odds Ratio
CI: Confidence Interval
WHO: World Health Organization
KNHANES: National Health and Nutrition Examination Survey
KDCA: Korea Centers for Disease Control and Prevention Agency
OECD: Organization for Economic Cooperation and Development
BMI: Body Mass Index
