# Supplementary figures and images for: Association between having a meal together with family and smoking: a cross-sectional nationwide survey
Source: BMC Public Health. 2023 Nov 16;23:2261. doi: 10.1186/s12889-023-17155-9 (PMC10655278; doi:10.1186/s12889-023-17155-9)

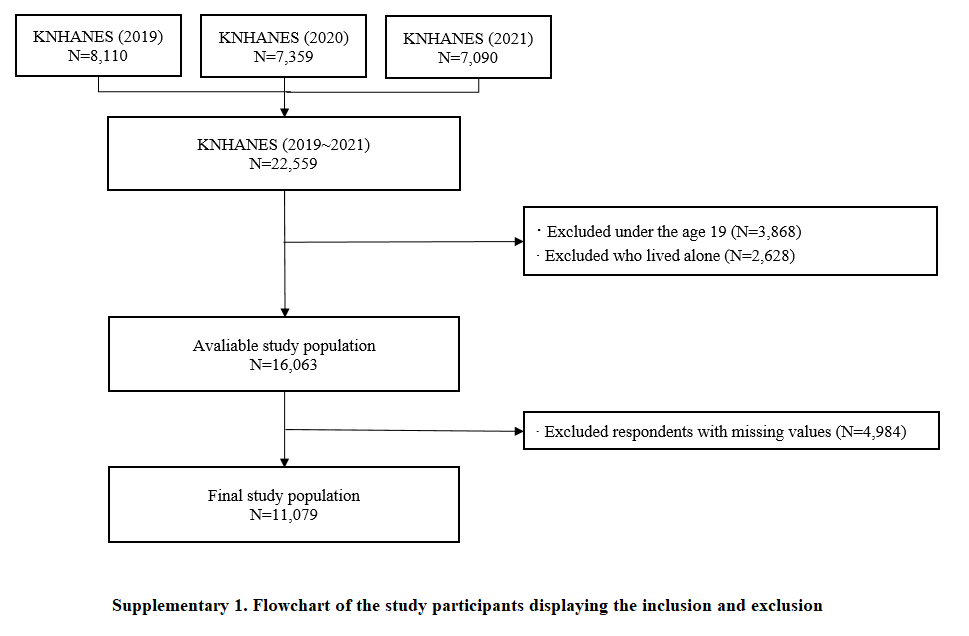

Supplement: Supplementary file 1 — Additional file 1: Supplementary 1. Flowchart of the study participants displaying the inclusion and exclusion. [file 12889_2023_17155_MOESM1_ESM.docx]
