# Supplementary material for: Association between having a meal together with family and smoking: a cross-sectional nationwide survey
Source: BMC Public Health. 2023 Nov 16;23:2261. doi: 10.1186/s12889-023-17155-9 (PMC10655278; doi:10.1186/s12889-023-17155-9)
Supplement: Supplementary file 2 — Additional file 2: Supplementary 2. General characteristics of the study population for each smoking behavior (cigarette type). [file 12889_2023_17155_MOESM2_ESM.docx]

| **Supplementary 2. General characteristics of the study population for each smoking behavior (cigarette type)** | | | | | | | | | | | | | | | | | | | | | | | | | | | |
| --- | --- | --- | --- | --- | --- | --- | --- | --- | --- | --- | --- | --- | --- | --- | --- | --- | --- | --- | --- | --- | --- | --- | --- | --- | --- | --- | --- |
| **Variables†** | **Smoking behavior (Cigarette type)** | | | | | | | | | | | | | | | | | | | | | | | | | | |
|  | **Male** | | | | | | | | | | | | ***P-value*** |  | **Female** | | | | | | | | | | | | ***P-value*** |
|  | **Total** | | **Never smoking** | | **Ex-smoking** | | **Only conventional** | | **Only electronic** | | **Dual smoking*** | |  |  | **Total** | | **Never smoking** | | **Ex-smoking** | | **Only electronic** | | **Only conventional** | | **Dual smoking*** | |  |
|  | **N** | **%** | **N** | **%** | **N** | **%** | **N** | **%** | **N** | **%** | **N** | **%** |  |  | **N** | **%** | **N** | **%** | **N** | **%** | **N** | **%** | **N** | **%** | **N** | **%** |  |
| **Total (N=11,079)** | 4,800 | 100.0 | 1,245 | 25.9 | 2,054 | 42.8 | 144 | 3.0 | 1,146 | 23.9 | 211 | 4.4 |  |  | 6,279 | 100.0 | 5,585 | 88.9 | 387 | 6.2 | 43 | 0.7 | 217 | 3.5 | 47 | 0.7 |  |
| **Having a meal together with family** |  |  |  |  |  |  |  |  |  |  |  |  | <.0001 |  |  |  |  |  |  |  |  |  |  |  |  |  | <.0001 |
| Yes | 3,855 | 80.3 | 961 | 24.9 | 1,752 | 45.4 | 114 | 3.0 | 875 | 22.7 | 153 | 4.0 |  |  | 5,211 | 83.0 | 4,686 | 89.9 | 316 | 6.1 | 32 | 0.6 | 147 | 2.8 | 30 | 0.6 |  |
| No | 945 | 19.7 | 284 | 30.1 | 302 | 32.0 | 30 | 3.2 | 271 | 28.7 | 58 | 6.1 |  |  | 1,068 | 17.0 | 899 | 84.2 | 71 | 6.6 | 11 | 1.0 | 70 | 6.6 | 17 | 1.6 |  |
| **Age** |  |  |  |  |  |  |  |  |  |  |  |  | <.0001 |  |  |  |  |  |  |  |  |  |  |  |  |  | <.0001 |
| 19-29 | 627 | 13.1 | 327 | 52.2 | 92 | 14.7 | 18 | 2.9 | 128 | 20.4 | 62 | 9.9 |  |  | 733 | 11.7 | 587 | 80.1 | 70 | 9.5 | 16 | 2.2 | 38 | 5.2 | 22 | 3.0 |  |
| 30-39 | 627 | 13.1 | 201 | 32.1 | 172 | 27.4 | 44 | 7.0 | 152 | 24.2 | 58 | 9.3 |  |  | 904 | 14.4 | 734 | 81.2 | 94 | 10.4 | 16 | 1.8 | 46 | 5.1 | 14 | 1.5 |  |
| 40-49 | 882 | 18.4 | 197 | 22.3 | 325 | 36.8 | 55 | 6.2 | 245 | 27.8 | 60 | 6.8 |  |  | 1,287 | 20.5 | 1,113 | 86.5 | 104 | 8.1 | 7 | 0.5 | 56 | 4.4 | 7 | 0.5 |  |
| 50-59 | 853 | 17.8 | 148 | 17.4 | 395 | 46.3 | 17 | 2.0 | 272 | 31.9 | 21 | 2.5 |  |  | 1,349 | 21.5 | 1,244 | 92.2 | 69 | 5.1 | 2 | 0.1 | 31 | 2.3 | 3 | 0.2 |  |
| 60- | 1,811 | 37.7 | 372 | 20.5 | 1,070 | 59.1 | 10 | 0.6 | 349 | 19.3 | 10 | 0.6 |  |  | 2,006 | 31.9 | 1,907 | 95.1 | 50 | 2.5 | 2 | 0.1 | 46 | 2.3 | 1 | 0.0 |  |
| **Marital status** |  |  |  |  |  |  |  |  |  |  |  |  | <.0001 |  |  |  |  |  |  |  |  |  |  |  |  |  | <.0001 |
| Married | 3,755 | 78.2 | 812 | 21.6 | 1,845 | 49.1 | 109 | 2.9 | 858 | 22.8 | 131 | 3.5 |  |  | 4,737 | 75.4 | 4,287 | 90.5 | 257 | 5.4 | 23 | 0.5 | 147 | 3.1 | 23 | 0.5 |  |
| Divorced, Separated | 937 | 19.5 | 425 | 45.4 | 177 | 18.9 | 31 | 3.3 | 227 | 24.2 | 77 | 8.2 |  |  | 1,266 | 20.2 | 1,084 | 85.6 | 95 | 7.5 | 17 | 1.3 | 48 | 3.8 | 22 | 1.7 |  |
| Single, widow | 108 | 2.3 | 8 | 7.4 | 32 | 29.6 | 4 | 3.7 | 61 | 56.5 | 3 | 2.8 |  |  | 276 | 4.4 | 214 | 77.5 | 35 | 12.7 | 3 | 1.1 | 22 | 8.0 | 2 | 0.7 |  |
| **Household income** |  |  |  |  |  |  |  |  |  |  |  |  | 0.0001 |  |  |  |  |  |  |  |  |  |  |  |  |  | 0.0152 |
| Low | 641 | 13.4 | 169 | 26.4 | 316 | 49.3 | 3 | 0.5 | 150 | 23.4 | 3 | 0.5 |  |  | 809 | 12.9 | 739 | 91.3 | 39 | 4.8 | 2 | 0.2 | 25 | 3.1 | 4 | 0.5 |  |
| Mid-low | 1,167 | 24.3 | 281 | 24.1 | 515 | 44.1 | 19 | 1.6 | 296 | 25.4 | 56 | 4.8 |  |  | 1,552 | 24.7 | 1,354 | 87.2 | 105 | 6.8 | 12 | 0.8 | 69 | 4.4 | 12 | 0.8 |  |
| Mid-high | 1,380 | 28.8 | 349 | 25.3 | 556 | 40.3 | 47 | 3.4 | 351 | 25.4 | 77 | 5.6 |  |  | 1,865 | 29.7 | 1,636 | 87.7 | 123 | 6.6 | 13 | 0.7 | 74 | 4.0 | 19 | 1.0 |  |
| High | 1,612 | 33.6 | 446 | 27.7 | 667 | 41.4 | 75 | 4.7 | 349 | 21.7 | 75 | 4.7 |  |  | 2,053 | 32.7 | 1,856 | 90.4 | 120 | 5.8 | 16 | 0.8 | 49 | 2.4 | 12 | 0.6 |  |
| **Educational level** |  |  |  |  |  |  |  |  |  |  |  |  | <.0001 |  |  |  |  |  |  |  |  |  |  |  |  |  | <.0001 |
| Middle school or below | 1,035 | 21.6 | 211 | 20.4 | 557 | 53.8 | 5 | 0.5 | 258 | 24.9 | 4 | 0.4 |  |  | 1,687 | 26.9 | 1,559 | 92.4 | 59 | 3.5 | 1 | 0.1 | 61 | 3.6 | 7 | 0.4 |  |
| High school | 1,714 | 35.7 | 435 | 25.4 | 664 | 38.7 | 44 | 2.6 | 475 | 27.7 | 96 | 5.6 |  |  | 2,130 | 33.9 | 1,816 | 85.3 | 157 | 7.4 | 25 | 1.2 | 111 | 5.2 | 21 | 1.0 |  |
| University or beyond | 2,051 | 42.7 | 599 | 29.2 | 833 | 40.6 | 95 | 4.6 | 413 | 20.1 | 111 | 5.4 |  |  | 2,462 | 39.2 | 2,210 | 89.8 | 171 | 6.9 | 17 | 0.7 | 45 | 1.8 | 19 | 0.8 |  |
| **Region** |  |  |  |  |  |  |  |  |  |  |  |  | 0.0032 |  |  |  |  |  |  |  |  |  |  |  |  |  | 0.0277 |
| Metropolitan | 2,043 | 42.6 | 509 | 24.9 | 869 | 42.5 | 69 | 3.4 | 490 | 24.0 | 106 | 5.2 |  |  | 2,835 | 45.2 | 2,510 | 88.5 | 180 | 6.3 | 28 | 1.0 | 93 | 3.3 | 24 | 0.8 |  |
| Urban | 1,737 | 36.2 | 480 | 27.6 | 728 | 41.9 | 60 | 3.5 | 396 | 22.8 | 73 | 4.2 |  |  | 2,285 | 36.4 | 2,022 | 88.5 | 151 | 6.6 | 13 | 0.6 | 80 | 3.5 | 19 | 0.8 |  |
| Rural | 1,020 | 21.3 | 256 | 25.1 | 457 | 44.8 | 15 | 1.5 | 260 | 25.5 | 32 | 3.1 |  |  | 1,159 | 18.5 | 1,053 | 90.9 | 56 | 4.8 | 2 | 0.2 | 44 | 3.8 | 4 | 0.3 |  |
| **Occupational categories** |  |  |  |  |  |  |  |  |  |  |  |  | <.0001 |  |  |  |  |  |  |  |  |  |  |  |  |  | 0.0003 |
| White | 1,367 | 28.5 | 394 | 28.8 | 560 | 41.0 | 77 | 5.6 | 253 | 18.5 | 83 | 6.1 |  |  | 1,500 | 23.9 | 1,315 | 87.7 | 112 | 7.5 | 10 | 0.7 | 46 | 3.1 | 17 | 1.1 |  |
| Pink | 489 | 10.2 | 121 | 24.7 | 171 | 35.0 | 25 | 5.1 | 131 | 26.8 | 41 | 8.4 |  |  | 954 | 15.2 | 823 | 86.3 | 70 | 7.3 | 10 | 1.0 | 40 | 4.2 | 11 | 1.2 |  |
| Blue | 1,584 | 33.0 | 336 | 21.2 | 700 | 44.2 | 25 | 1.6 | 475 | 30.0 | 48 | 3.0 |  |  | 890 | 14.2 | 817 | 91.8 | 30 | 3.4 | 2 | 0.2 | 37 | 4.2 | 4 | 0.4 |  |
| Inoccupation | 1,360 | 28.3 | 394 | 29.0 | 623 | 45.8 | 17 | 1.3 | 287 | 21.1 | 39 | 2.9 |  |  | 2,935 | 46.7 | 2,630 | 89.6 | 175 | 6.0 | 21 | 0.7 | 94 | 3.2 | 15 | 0.5 |  |
| **Physical activity** |  |  |  |  |  |  |  |  |  |  |  |  | <.0001 |  |  |  |  |  |  |  |  |  |  |  |  |  | 0.1424 |
| Adequate | 2,221 | 46.3 | 642 | 28.9 | 924 | 41.6 | 64 | 2.9 | 476 | 21.4 | 115 | 5.2 |  |  | 2,568 | 40.9 | 2,294 | 89.3 | 158 | 6.2 | 14 | 0.5 | 77 | 3.0 | 25 | 1.0 |  |
| Inadequate | 2,579 | 53.7 | 603 | 23.4 | 1,130 | 43.8 | 80 | 3.1 | 670 | 26.0 | 96 | 3.7 |  |  | 3,711 | 59.1 | 3,291 | 88.7 | 229 | 6.2 | 29 | 0.8 | 140 | 3.8 | 22 | 0.6 |  |
| **Current drinking status** |  |  |  |  |  |  |  |  |  |  |  |  | <.0001 |  |  |  |  |  |  |  |  |  |  |  |  |  | <.0001 |
| Never or occasionally | 958 | 20.0 | 336 | 35.1 | 451 | 47.1 | 12 | 1.3 | 149 | 15.6 | 10 | 1.0 |  |  | 2,231 | 35.5 | 2,093 | 93.8 | 83 | 3.7 | 5 | 0.2 | 49 | 2.2 | 1 | 0.0 |  |
| 2~4 times / month | 2,320 | 48.3 | 700 | 30.2 | 899 | 38.8 | 80 | 3.4 | 521 | 22.5 | 120 | 5.2 |  |  | 3,373 | 53.7 | 2,988 | 88.6 | 228 | 6.8 | 24 | 0.7 | 102 | 3.0 | 31 | 0.9 |  |
| 2~4 times / week | 1,522 | 31.7 | 209 | 13.7 | 704 | 46.3 | 52 | 3.4 | 476 | 31.3 | 81 | 5.3 |  |  | 675 | 10.8 | 504 | 74.7 | 76 | 11.3 | 14 | 2.1 | 66 | 9.8 | 15 | 2.2 |  |
| **BMI** |  |  |  |  |  |  |  |  |  |  |  |  | 0.0092 |  |  |  |  |  |  |  |  |  |  |  |  |  | 0.0141 |
| Normal and underweight | 1,463 | 30.5 | 402 | 27.5 | 592 | 40.5 | 33 | 2.3 | 380 | 26.0 | 56 | 3.8 |  |  | 3,134 | 49.9 | 2,778 | 88.6 | 207 | 6.6 | 29 | 0.9 | 94 | 3.0 | 26 | 0.8 |  |
| Overweight | 1,265 | 26.4 | 312 | 24.7 | 571 | 45.1 | 34 | 2.7 | 298 | 23.6 | 50 | 4.0 |  |  | 1,282 | 20.4 | 1,153 | 89.9 | 76 | 5.9 | 4 | 0.3 | 38 | 3.0 | 11 | 0.9 |  |
| Obese | 2,072 | 43.2 | 531 | 25.6 | 891 | 43.0 | 77 | 3.7 | 468 | 22.6 | 105 | 5.1 |  |  | 1,863 | 29.7 | 1,654 | 88.8 | 104 | 5.6 | 10 | 0.5 | 85 | 4.6 | 10 | 0.5 |  |
| **Number of chronic diseases** |  |  |  |  |  |  |  |  |  |  |  |  | <.0001 |  |  |  |  |  |  |  |  |  |  |  |  |  | <.0001 |
| 0 | 2,372 | 49.4 | 757 | 31.9 | 822 | 34.7 | 89 | 3.8 | 560 | 23.6 | 144 | 6.1 |  |  | 3,512 | 55.9 | 3,031 | 86.3 | 271 | 7.7 | 39 | 1.1 | 129 | 3.7 | 42 | 1.2 |  |
| 1 | 1,454 | 30.3 | 310 | 21.3 | 696 | 47.9 | 34 | 2.3 | 367 | 25.2 | 47 | 3.2 |  |  | 1,598 | 25.4 | 1,467 | 91.8 | 73 | 4.6 | 3 | 0.2 | 51 | 3.2 | 4 | 0.3 |  |
| ≥2 | 974 | 20.3 | 178 | 18.3 | 536 | 55.0 | 21 | 2.2 | 219 | 22.5 | 20 | 2.1 |  |  | 1,169 | 18.6 | 1,087 | 93.0 | 43 | 3.7 | 1 | 0.1 | 37 | 3.2 | 1 | 0.1 |  |
| **Household generation composition** |  |  |  |  |  |  |  |  |  |  |  |  | <.0001 |  |  |  |  |  |  |  |  |  |  |  |  |  | 0.0004 |
| 1st generation | 1,662 | 34.6 | 369 | 22.2 | 890 | 53.5 | 23 | 1.4 | 339 | 20.4 | 41 | 2.5 |  |  | 1,789 | 28.5 | 1,632 | 91.2 | 72 | 4.0 | 11 | 0.6 | 62 | 3.5 | 12 | 0.7 |  |
| 2nd generation | 2,876 | 59.9 | 824 | 28.7 | 1,048 | 36.4 | 114 | 4.0 | 737 | 25.6 | 153 | 5.3 |  |  | 4,024 | 64.1 | 3,552 | 88.3 | 281 | 7.0 | 29 | 0.7 | 129 | 3.2 | 33 | 0.8 |  |
| 3rd generation or more | 262 | 5.5 | 52 | 19.8 | 116 | 44.3 | 7 | 2.7 | 70 | 26.7 | 17 | 6.5 |  |  | 466 | 7.4 | 401 | 86.1 | 34 | 7.3 | 3 | 0.6 | 26 | 5.6 | 2 | 0.4 |  |
| **Number of household members** |  |  |  |  |  |  |  |  |  |  |  |  | <.0001 |  |  |  |  |  |  |  |  |  |  |  |  |  | 0.0069 |
| 2 | 1,920 | 40.0 | 449 | 23.4 | 953 | 49.6 | 31 | 1.6 | 436 | 22.7 | 51 | 2.7 |  |  | 2,325 | 37.0 | 2,086 | 89.7 | 119 | 5.1 | 13 | 0.6 | 92 | 4.0 | 15 | 0.6 |  |
| 3 | 1,354 | 28.2 | 379 | 28.0 | 526 | 38.8 | 52 | 3.8 | 336 | 24.8 | 61 | 4.5 |  |  | 1,815 | 28.9 | 1,612 | 88.8 | 106 | 5.8 | 14 | 0.8 | 67 | 3.7 | 16 | 0.9 |  |
| 4 | 1,147 | 23.9 | 321 | 28.0 | 430 | 37.5 | 48 | 4.2 | 278 | 24.2 | 70 | 6.1 |  |  | 1,562 | 24.9 | 1,390 | 89.0 | 116 | 7.4 | 13 | 0.8 | 32 | 2.0 | 11 | 0.7 |  |
| ≥5 | 379 | 7.9 | 96 | 25.3 | 145 | 38.3 | 13 | 3.4 | 96 | 25.3 | 29 | 7.7 |  |  | 577 | 9.2 | 497 | 86.1 | 46 | 8.0 | 3 | 0.5 | 26 | 4.5 | 5 | 0.9 |  |
| **Frequency of eating out** |  |  |  |  |  |  |  |  |  |  |  |  | <.0001 |  |  |  |  |  |  |  |  |  |  |  |  |  | <.0001 |
| Everyday | 1,367 | 28.5 | 386 | 28.2 | 485 | 35.5 | 59 | 4.3 | 343 | 25.1 | 94 | 6.9 |  |  | 861 | 13.7 | 752 | 87.3 | 54 | 6.3 | 7 | 0.8 | 37 | 4.3 | 11 | 1.3 |  |
| 1 times more / week | 2,335 | 48.6 | 617 | 26.4 | 995 | 42.6 | 71 | 3.0 | 544 | 23.3 | 108 | 4.6 |  |  | 3,339 | 53.2 | 2,897 | 86.8 | 260 | 7.8 | 31 | 0.9 | 116 | 3.5 | 35 | 1.0 |  |
| 1 times more / month | 785 | 16.4 | 175 | 22.3 | 401 | 51.1 | 11 | 1.4 | 192 | 24.5 | 6 | 0.8 |  |  | 1,496 | 23.8 | 1,398 | 93.4 | 53 | 3.5 | 3 | 0.2 | 41 | 2.7 | 1 | 0.1 |  |
| Never or less than once a month | 313 | 6.5 | 67 | 21.4 | 173 | 55.3 | 3 | 1.0 | 67 | 21.4 | 3 | 1.0 |  |  | 583 | 9.3 | 538 | 92.3 | 20 | 3.4 | 2 | 0.3 | 23 | 3.9 | 0 | 0.0 |  |
| **Year** |  |  |  |  |  |  |  |  |  |  |  |  | 0.1627 |  |  |  |  |  |  |  |  |  |  |  |  |  | 0.6099 |
| 2019 | 1,840 | 38.3 | 455 | 24.7 | 786 | 42.7 | 56 | 3.0 | 443 | 24.1 | 100 | 5.4 |  |  | 2,406 | 38.3 | 2,131 | 88.6 | 160 | 6.7 | 13 | 0.5 | 79 | 3.3 | 23 | 1.0 |  |
| 2020 | 1,482 | 30.9 | 402 | 27.1 | 615 | 41.5 | 46 | 3.1 | 365 | 24.6 | 54 | 3.6 |  |  | 1,940 | 30.9 | 1,728 | 89.1 | 114 | 5.9 | 13 | 0.7 | 72 | 3.7 | 13 | 0.7 |  |
| 2021 | 1,478 | 30.8 | 388 | 26.3 | 653 | 44.2 | 42 | 2.8 | 338 | 22.9 | 57 | 3.9 |  |  | 1,933 | 30.8 | 1,726 | 89.3 | 113 | 5.8 | 17 | 0.9 | 66 | 3.4 | 11 | 0.6 |  |
| † All variables are categorized variables, and descriptive statistics are presented as frequency (N) and percentage (%) * Dual smoking means people who use conventional and electronic cigarettes | | | | | | | | | | | | | | | | | | | | | | | | | | | |
