# Supplementary material for: Association between having a meal together with family and smoking: a cross-sectional nationwide survey
Source: BMC Public Health. 2023 Nov 16;23:2261. doi: 10.1186/s12889-023-17155-9 (PMC10655278; doi:10.1186/s12889-023-17155-9)
Supplement: Supplementary file 3 — Additional file 3: Supplementary 3. Subgroup analysis of smoking cessation attempt and plan among all participants. [file 12889_2023_17155_MOESM3_ESM.docx]

| **Supplementary 3. Subgroup analysis of smoking cessation attempt and plan among all participants** | | | | | | | | | | |
| --- | --- | --- | --- | --- | --- | --- | --- | --- | --- | --- |
| **Variables†** | **Having a meal together with family (Ref = 'Yes')** | | | | | | | | | |
|  | **Male** | | | |  | **Female** | | | | |
|  | **OR** | **95% CI** | | |  | **OR** | **95% CI** | | | |
| **Smoking cessation attempt** |  |  |  |  |  |  |  |  |  |  |
| Never smoking | 1.00 |  |  |  |  | 1.00 |  |  |  |  |
| Previously smoking | 0.94 | (0.73 | - | 1.21) |  | 1.06 | (0.73 | - | 1.53) |  |
| Currently smoking (Yes) | 1.18 | (0.89 | - | 1.56) |  | 1.78 | (1.00 | - | 3.17) |  |
| Currently smoking (No) | 1.25 | (0.93 | - | 1.69) |  | 2.02 | (1.33 | - | 3.06) |  |
| **Smoking cessation plan** |  |  |  |  |  |  |  |  |  |  |
| Never smoking | 1.00 |  |  |  |  | 1.00 |  |  |  |  |
| Previously smoking | 0.94 | (0.73 | - | 1.21) |  | 1.06 | (0.73 | - | 1.53) |  |
| Currently smoking (Yes) | 1.09 | (0.83 | - | 1.44) |  | 1.76 | (1.12 | - | 2.75) |  |
| Currently smoking (No) | 1.50 | (1.09 | - | 2.07) |  | 2.38 | (1.42 | - | 4.00) |  |
| † Adjusted for all covariates (age, marital status, educational level, region, occupational categories, household generation composition, and number of household members) | | | | | | | | | | |
